# The Cottage Hospital, Staines

**Published:** 1914-10-03

**Authors:** Leslie Moore

**Affiliations:** 3 Raymond Buildings, Gray's Inn, W.C.


*To the Editor of* The Hospital.

### The Cottage Hospital, Staines.

*To the Editor of* The Hospital.

Sir,—By chance your account of the Cottage Hospital at Staines in the current number of The Hospital has been brought to my notice. I note the remark that "No provision beyond an ordinary sink appears to be made for emptying or cleansing bed pans." I shall be glad if you will correct in your next issue this erroneous statement, for a proper combination hospital sink for washing bed pans and urine bottles, with flushing rim and jets, is provided.

Having had special tracings made for your use I think the least you could do in return would have been to refer to me before giving such incorrect impressions if you did not intend to visit the building.

Your hint that there was no skilled advice obtained in assessing the competition is quite unfounded.—Yours faithfully,

Leslie Moore.

3 Raymond Buildings, Gray's Inn, W.C.,
September 29, 1914.

[We have pleasure in publishing Mr. Moore's letter. A copy of The Hospital was sent to him in the ordinary course. The plan gives no indication of the existence of a bed-pan sink. If, as must be inferred from Mr. Moore's letter, it is placed in the patients' w.c. the arrangement is not a good one—a separate sink-room should have been provided. The absence of such a sink-room on the plan was not unreasonably taken as indicating that it did not exist. Otherwise we should have raised the point, as is our practice, with the architect.—Ed. The Hospital.]

